# Cervico-suboccipital sensorimotor convergence as an upstream autonomic pathway in bilateral rhythmic stimulation

**DOI:** 10.3389/fnint.2026.1862966

**Published:** 2026-08-24

**Authors:** Ioulia Milovanov

**Affiliations:** Private Practitioner, Tel-Aviv, Israel

**Keywords:** autonomic regulation, bilateral rhythmic stimulation, cerebrospinal fluid dynamics, cervico-suboccipital system, eye movement desensitization and reprocessing, meningeal lymphatic drainage, neuroimmune modulation, sensorimotor convergence

## Abstract

Rhythmic bilateral stimulation is a defining procedural feature of Eye Movement Desensitization and Reprocessing (EMDR), yet its physiological significance remains unresolved. Existing accounts have mainly emphasized rapid cognitive and attentional mechanisms during traumatic memory processing, including working-memory taxation, dual attention, and reductions in memory vividness and emotionality. This Hypothesis and Theory article develops a complementary upstream physiological model that separates acute cognitive-attentional effects from proximal sensorimotor-autonomic coupling and slower downstream neuroimmune hypotheses. It proposes that bilateral or spatially organized rhythmic stimulation generates modality-specific ocular, vestibulo-cervical, proprioceptive, autonomic, and cervico-suboccipital signatures. These signatures may contribute to rapid bodily-autonomic effects, such as orienting responses and parasympathetic shifts, through brainstem integration and vagal efferent output, without requiring direct vagal afferent stimulation from the stimulated site. Across repeated stimulation, the resulting autonomic-vascular state is proposed to interface with cerebrospinal fluid dynamics, meningeal lymphatic exchange, deep cervical lymph node drainage, and glial or regulatory immune mechanisms, including the regulatory T-cell-centered pathway proposed previously. Mastoid and peri-auricular stimulation are discussed as a potentially shorter upper-cervical entry point to this sensorimotor-autonomic pathway because their anatomical position is suited to engage local somatosensory, vestibular-adjacent, cervical proprioceptive, and cervico-suboccipital systems. The model is presented as a testable physiological framework that requires direct experimental validation. It generates predictions for future studies combining standardized rhythmic stimulation protocols with ocular, cervical, autonomic, cerebrospinal fluid, meningeal lymphatic, and neuroimmune measures.

## 1 Introduction

Eye Movement Desensitization and Reprocessing (EMDR) is an established psychotherapeutic approach in which emotionally distressing or traumatic memories are processed while the individual receives rhythmic bilateral stimulation. The original procedure was developed around guided eye movements (Shapiro, 1989), but tactile, auditory, vibrotactile, and other forms of alternating bilateral stimulation are now widely used in clinical and self-regulatory contexts. This diversity of stimulation modalities raises a mechanistic question that remains incompletely resolved: are the effects of bilateral stimulation explained primarily by general cognitive load, or does the spatial and rhythmic structure of stimulation also generate distinct physiological signatures?

Several mechanisms have been proposed to explain EMDR and alternating bilateral stimulation. Cognitive and memory-based accounts emphasize working-memory taxation, dual attention, reduced vividness and emotionality of traumatic imagery, and changes in memory retrieval or reconsolidation (Schubert and Lee, 2009; van den Hout and Engelhard, 2012; Landin-Romero et al., 2018). Other accounts emphasize orienting responses, autonomic regulation, interhemispheric interaction, REM-like processes, or changes in arousal and emotional processing (Sack et al., 2008; Jeffries and Davis, 2013). Stochastic resonance has also been proposed as a neurobiological model in which alternating bilateral stimulation may act as a structured sensory signal that facilitates adaptive information processing in nonlinear neural systems (Miller et al., 2018). These mechanisms are not mutually exclusive. EMDR is likely to involve interacting cognitive, affective, autonomic, sensorimotor, and neurobiological processes rather than a single isolated pathway.

A central unresolved issue is whether the spatial and rhythmic structure of stimulation contributes effects beyond general dual-task load. In the EMDR literature, this question has often been framed around the specific contribution of eye movements. Working-memory accounts suggest that reductions in memory vividness and emotionality can be produced by tasks that sufficiently tax working memory, including vertical eye movements or non-ocular dual tasks, indicating that strictly horizontal or bilateral stimulation may not be necessary for these rapid cognitive effects (van den Hout and Engelhard, 2012). In contrast, Lee and Cuijpers (2013) reported a significant additional effect of eye movements in both treatment and laboratory studies, whereas Jeffries and Davis (2013) concluded that clinical evidence remains contradictory and analogue studies provide more positive support. Thus, the specificity and necessity of eye movements or bilateral stimulation remain unresolved.

The present hypothesis therefore distinguishes spatial alternation from strict left–right specificity. It does not challenge the working-memory account of rapid memory effects. Instead, it asks whether rhythmic spatially organized stimulation, including but not limited to alternating left–right stimulation, can generate ocular, vestibulo-cervical, proprioceptive, autonomic, and cervico-suboccipital signatures that have not been captured by studies focusing primarily on memory vividness and emotionality. One plausible reason why spatial alternation matters is proprioceptive novelty and reduced habituation: alternating spatial input repeatedly shifts the pattern of ocular, cervical, tactile, vestibular, and autonomic engagement, thereby maintaining sensorimotor salience more effectively than monotonic or simultaneous stimulation. Habituation is characteristically stimulus-specific and depends on repeated exposure to the same stimulus pattern (Rankin et al., 2009).

The relevance of spatial alternation in the present model is therefore primarily sensorimotor and autonomic rather than mechanical. Alternating stimulation repeatedly changes the spatial source of sensory input, which may maintain orienting, ocular preparation, cervical proprioceptive salience, and autonomic responsiveness more effectively than monotonic or simultaneous stimulation. This does not imply that horizontal left–right stimulation is uniquely required for rapid EMDR effects. Rather, spatial organization is treated as an experimental parameter that may shape the bodily signature of rhythmic stimulation.

This distinction is important because EMDR is not treated here as the only or exclusive carrier of the proposed mechanism. EMDR provides a clinically important context in which rhythmic bilateral stimulation is widely used. However, the broader question concerns bilateral or spatially organized rhythmic stimulation as a physiological principle. From this perspective, guided eye movements, alternating tactile stimulation, alternating auditory stimulation, vibrotactile stimulation, and mastoid or peri-auricular stimulation differ not only in their cognitive load, but also in the sensorimotor, cervical, vestibular, autonomic, and cervico-suboccipital signatures they generate.

A previously proposed neuroimmune model hypothesized that bilateral rhythmic stimulation influences autonomic balance, cerebrospinal fluid dynamics, meningeal lymphatic function, regulatory immune mechanisms, microglial activity, and neuroinflammatory tone (Milovanov, 2026). That model focused mainly on downstream physiological and neuroimmune processes that follow or accompany repeated bilateral stimulation. However, an upstream question remained insufficiently addressed: through what sensorimotor or autonomic route might different forms of bilateral stimulation initiate or facilitate such physiological effects?

The present article proposes one possible upstream route: cervico-suboccipital sensorimotor convergence. According to this hypothesis, visual, tactile, auditory, vibrotactile, vestibular-adjacent, or peri-auricular stimulation each engages, through different sensory routes, repeated orienting, gaze-postural preparation, cervical proprioceptive regulation, and brainstem-autonomic integration. In visually guided EMDR, this pathway is engaged relatively directly through repeated eye movements, gaze shifts, and eye-head–neck coordination. In non-visual bilateral stimulation, the route is less direct and involves covert spatial orienting, microsaccadic modulation, ocular motor preparation, somatosensory orienting, vestibulo-cervical coupling, proprioceptive regulation, or autonomic shifts.

The hypothesis should therefore not be understood as claiming that every form of bilateral stimulation necessarily produces overt saccades or visible head movement. A more cautious formulation is that spatially organized rhythmic stimulation biases the organism toward repeated orienting and gaze-postural preparation. This includes ocular preparation, microsaccadic activity, cervical proprioceptive adjustments, vestibulo-cervical coupling, and subtle changes in suboccipital recruitment or tone. The magnitude of these effects is expected to depend on stimulation modality, stimulation frequency, spatial salience, posture, fixation demands, baseline autonomic state, and individual sensorimotor organization.

The anatomical plausibility of this proposal is supported by the role of the suboccipital and upper cervical region in fine head-position control, gaze stabilization, vestibular integration, and proprioceptive monitoring. Suboccipital muscles have a high density of muscle spindles and are well suited to provide continuous information about head position and small changes in cervical state (Kulkarni et al., 2001). In the present model, this region is treated primarily as a proprioceptive and sensorimotor convergence interface rather than as a direct mechanical driver of cerebrospinal fluid flow.

Mastoid and peri-auricular stimulation are discussed in this article as a potentially shorter experimental route to the same cervico-suboccipital sensorimotor-autonomic pathway. Stimulation near the mastoid or peri-auricular region is anatomically relevant because it is close to local soft tissues, the temporal bone, vestibular-adjacent structures, cervical proprioceptive systems, and suboccipital regulation. For this reason, it can help test whether rhythmic spatial stimulation produces measurable cervico-suboccipital and autonomic signatures more directly than distal tactile or auditory routes. In this sense, mastoid or peri-auricular stimulation is not a departure from the broader bilateral stimulation framework, but a more anatomically targeted way to examine one of its possible physiological entry points.

The model developed here is therefore deliberately limited but testable. It does not reduce EMDR to a bodily mechanism and does not replace cognitive, memory-related, orienting, autonomic, or stochastic resonance accounts. Instead, it adds an upstream physiological layer: repeated spatially organized rhythmic stimulation may engage a cervico-suboccipital sensorimotor-autonomic pathway that interfaces, over a slower timescale, with vascular, cerebrospinal fluid, meningeal lymphatic, and neuroimmune regulation.

The article develops this cervico-suboccipital sensorimotor convergence hypothesis as an upstream extension of the previously proposed neuroimmune model. It outlines the sensorimotor and anatomical rationale, distinguishes acute cognitive and bodily-autonomic effects from slower repeated-stimulation mechanisms, integrates the hypothesis with cerebrospinal fluid, meningeal lymphatic, and regulatory immune pathways, and proposes experimental strategies for testing the model using eye tracking, microsaccade analysis, cervical electromyography, head–neck micro-motion analysis, autonomic monitoring, and indirect or translational measures of cerebrospinal fluid and lymphatic function.

## 2 Integrative sensorimotor-autonomic model

The model proposed here begins with a distinction between three levels of bilateral or spatially organized rhythmic stimulation. The first is a rapid cognitive-attentional level, involving working-memory taxation, dual attention, and changes in the vividness and emotionality of activated memories. This level is especially relevant to EMDR when traumatic memory processing is central. The second is a rapid bodily-autonomic level, involving orienting responses, ocular preparation, vestibulo-cervical pathways, proprioceptive input, brainstem autonomic regulation, and possible vagal efferent parasympathetic shifts. This level may contribute to acute bodily changes such as reduced arousal, relaxation, or a subjective sense of release. The third is a slower bodily-neuroimmune level, in which repeated sensorimotor-autonomic and autonomic-vascular modulation may interact with cerebrospinal fluid dynamics, meningeal lymphatic exchange, deep cervical lymph node drainage, glial regulation, and regulatory immune tone.

These levels are not presented as competing explanations. They can operate in parallel, and their relative contribution depends on the stimulation modality, clinical context, task instructions, individual autonomic state, posture, and whether traumatic memory processing is occurring. This separation is important because mechanisms that explain rapid changes during a single session are not necessarily the same as mechanisms that operate through repeated stimulation across days or weeks.

The central question is therefore whether rhythmic spatial stimulation can produce a measurable sensorimotor and cervico-suboccipital-autonomic signature. If such a signature exists, it could provide an upstream route through which repeated stimulation interacts with cerebrospinal fluid, meningeal lymphatic, and neuroimmune processes over a slower timescale.

### 2.1 Bilateral rhythmic stimulation as a multimodal physiological input

Bilateral stimulation is often described as an alternating sensory signal. However, alternating sensory input is not only perceptual. It can also induce orienting responses, attentional shifts, gaze preparation, postural adjustments, autonomic changes, and small sensorimotor responses. These responses may occur even when the person remains still and no overt movement is visible.

Visual bilateral stimulation engages eye movement systems, gaze orientation, and eye-head coordination. Tactile bilateral stimulation engages somatosensory orienting and proprioceptive regulation. Auditory bilateral stimulation engages spatial attention, acoustic orienting, and postural preparation. Vibrotactile or peri-auricular stimulation additionally recruits local mechanoreceptive, vestibular-adjacent, cervical, or autonomic pathways.

Thus, the relevant question is not whether visual, tactile, auditory, and vibratory stimulation are identical. They are not. The question is whether they may partly converge on shared rhythmic sensorimotor and autonomic systems. The cervico-suboccipital region is a plausible candidate convergence site because it participates in fine head-position control, gaze stabilization, proprioceptive regulation, vestibular integration, and cranio-cervical sensorimotor coupling.

In this framework, bilateral stimulation is treated as a family of spatially organized rhythmic inputs. Different forms of stimulation enter the nervous system through different sensory channels, but they can still bias the organism toward repeated orienting, gaze-postural preparation, and subtle cervical adjustment.

### 2.2 Rapid cognitive and bodily-autonomic effects

A distinction should be made between rapid cognitive effects and rapid bodily-autonomic effects. In EMDR, rapid reductions in memory vividness and emotionality may be explained by working-memory taxation, dual attention, or other memory-related mechanisms, particularly when the therapeutic procedure requires the person to hold a traumatic or emotionally salient memory in mind while performing a concurrent task (van den Hout and Engelhard, 2012; Landin-Romero et al., 2018).

However, not all rapid effects of rhythmic stimulation are necessarily cognitive in this narrow sense. Alternating or spatially organized stimulation may also produce fast bodily-autonomic effects, including orienting responses, changes in arousal, shifts in respiration, changes in heart rate variability, pupillary responses, electrodermal changes, or subjective sensations of relaxation and release (Sack et al., 2008).

This bodily-autonomic level is important because it provides a rapid physiological pathway that is separate from the slower cerebrospinal fluid and lymphatic hypothesis. Rhythmic spatial stimulation may engage orienting networks, ocular preparation, vestibulo-cervical pathways, cervical proprioception, and brainstem autonomic circuits. These pathways may contribute to acute calming or regulation while also shaping the physiological state in which slower repeated-session effects could occur.

### 2.3 Cervico-ocular and vestibulo-cervical coupling

Eye movements are embedded within an integrated eye-head–neck system that supports orientation toward sensory stimuli, gaze stabilization, and head-position regulation. Even when the head does not visibly move, preparation for visual orientation and saccadic shifts can be accompanied by activity in neck muscles (Corneil et al., 2004). This suggests that visual bilateral stimulation includes a subtle cervical motor component in addition to its ocular and cognitive components.

The hypothesis proposed here does not require large head movements or voluntary neck contractions. It requires only measurable stimulation-related changes in cervical or suboccipital recruitment, tone, proprioceptive afference, or head–neck micro-motion. Such changes are primarily relevant here as sensorimotor and autonomic signals, not as proof of a mechanically significant displacement of cerebrospinal fluid.

Vestibulo-cervical pathways provide another possible route. Vestibular input is closely connected to cervical muscle activity and postural regulation. Stimulation near the mastoid or peri-auricular region can engage vestibular-adjacent or cervico-vestibular pathways more directly than distal tactile stimulation. Cervical vestibular-evoked myogenic potential studies and related vestibular stimulation paradigms show that vestibular and bone-conducted stimuli can evoke short-latency responses in neck musculature (Rosengren et al., 2010; Colebatch et al., 2016; Forbes et al., 2018). Similarly, auditory bilateral stimulation can engage spatial orienting and head–neck preparation even in the absence of overt movement.

This does not imply that all stimulation modalities produce the same cervical response. Visual stimulation, auditory stimulation, distal tactile stimulation, vibrotactile stimulation, and mastoid-region stimulation differ in the strength, timing, and anatomical route of their sensorimotor effects. The point is more limited: each modality has a direct or indirect route through which it can influence the eye-head–neck system.

### 2.4 Covert ocular orienting and microsaccadic modulation

A particularly important possibility is that non-visual bilateral stimulation can recruit the oculomotor system through covert spatial orienting. Alternating tactile, auditory, or vibrotactile stimuli shift spatial attention from side to side even when the eyes remain apparently still. Such covert attentional shifts can be accompanied by microsaccadic modulation, subtle gaze tendencies, or ocular motor preparation toward the stimulated side. Microsaccades have been proposed as an overt marker of covert attentional shifts, and spatial cueing paradigms show that microsaccade rate and direction can be modulated by covert attention (Hafed and Clark, 2002; Engbert and Kliegl, 2003; Laubrock et al., 2005).

This provides a possible bridge between visual and non-visual forms of bilateral stimulation. In visually guided bilateral stimulation, the oculomotor system is engaged directly through eye movements. In tactile, auditory, or vibrotactile bilateral stimulation, the oculomotor system can be engaged indirectly through spatial attention and orienting mechanisms. Because ocular motor preparation is coupled with head–neck motor circuits, covert ocular orienting provides one route through which non-visual bilateral stimulation influences cervical and suboccipital muscle tone.

This link should be interpreted as a testable possibility rather than an obligatory response. The relationship between covert attention, microsaccades, and saccade preparation is context-dependent (Casteau and Smith, 2019; Liu et al., 2024). Non-visual bilateral stimulation will not necessarily produce measurable microsaccadic modulation in every paradigm or every individual. Fixation instructions, sensory salience, stimulation frequency, task demands, anxiety level, and baseline oculomotor state can all influence the result.

In this framework, the eye-neck-suboccipital axis becomes a possible convergence system for different bilateral stimulation modalities. Visual bilateral stimulation may activate this axis explicitly through eye movements, whereas non-visual stimulation may activate it implicitly through covert orienting, microsaccadic modulation, or premotor gaze preparation. The key claim is not that every bilateral stimulus produces overt saccades, but that spatially organized rhythmic stimulation may bias the organism toward repeated orienting and gaze-postural preparation that can be tested with eye tracking, cervical electromyography, and head–neck micro-motion analysis.

### 2.5 Suboccipital muscles as a proprioceptive convergence interface

The suboccipital muscles are small deep muscles located at the cranio-cervical junction. They contribute to fine control of head position, proprioception, and stabilization of the upper cervical region. Their anatomical position makes them especially relevant to interactions among eye movement, head orientation, vestibular processing, and cervical proprioception.

The suboccipital muscles are relevant not only because of their anatomical relationship to the cranio-cervical junction, but also because they form a dense proprioceptive interface. Quantitative anatomical work has shown a high density of muscle spindles in human suboccipital muscles, supporting their role in fine head-position monitoring and eye-head coordination (Kulkarni et al., 2001). This sensory role is important for the present model. The proposed rapid sensorimotor and autonomic effects do not require large-amplitude head or neck movement. Even low-amplitude or subthreshold changes in suboccipital tone, tension, or recruitment may be sufficient to modulate proprioceptive afference from this region.

Cervico-ocular and eye-head coupling literature supports the idea that ocular orienting and saccade preparation can be accompanied by cervical or neck-muscle modulation even in the absence of overt head movement (Corneil et al., 2004). In the present model, such activation is treated as a proprioceptive and autonomic signal that reports eye-head–neck state.

For the present model, the importance of this region is therefore not that it could mechanically transmit force to deeper tissues, but that it provides a dense proprioceptive interface linked to gaze, balance, head position, and autonomic state. Suboccipital recruitment can be investigated as a physiological signature of orienting and eye-head–neck coordination, even when no visible movement occurs.

This narrows the hypothesis. The immediate claim is that rhythmic spatial stimulation may organize ocular, cervical, suboccipital, and autonomic activity together; any downstream cerebrospinal fluid, vascular, lymphatic, or neuroimmune effects are treated as later experimental targets rather than immediate consequences of suboccipital activation.

The critical step for future research is therefore to determine whether rhythmic stimulation produces measurable suboccipital or upper cervical signatures, whether those signatures are coupled to autonomic regulation, and whether repeated stimulation-related autonomic-vascular states are associated with downstream cerebrospinal fluid, lymphatic, glial, or immune measures.

### 2.6 The cervico-suboccipital sensorimotor convergence hypothesis

The central proposal of this article is that bilateral or spatially organized rhythmic stimulation can converge on a cervico-suboccipital sensorimotor-autonomic interface at the cranio-cervical junction. This convergence is hypothesized to represent a functional interaction among bilateral sensory input, spatial orienting, ocular preparation, cervical proprioceptive regulation, vestibulo-cervical coupling, suboccipital recruitment, and brainstem-autonomic integration.

The term convergence is used deliberately. It does not imply visible head motion or strong muscle contraction; rather, it refers to the possibility that different sensory modalities, although entering through different peripheral channels, partially converge on shared orienting, gaze-postural, upper cervical, and autonomic circuits.

The hypothesized pathway can be described as a staged sensorimotor-autonomic sequence rather than a simple linear mechanical chain. Rhythmic spatial stimulation repeatedly engages orienting, ocular, vestibulo-cervical, proprioceptive, and autonomic systems. These systems influence the cervico-suboccipital region by modulating proprioceptive afference, cervical recruitment, head–neck micro-motion, and brainstem autonomic integration. Over repeated stimulation, the resulting autonomic-vascular state could interact with cerebrospinal fluid exchange, meningeal lymphatic drainage, deep cervical lymph node immune surveillance, and regulatory immune processes.

This formulation identifies a set of experimentally separable links. The first is whether rhythmic stimulation produces ocular, cervical, suboccipital, or autonomic signatures. The second is whether these signatures differ across visual, tactile, auditory, vibrotactile, and mastoid or peri-auricular stimulation. The third is whether such signatures are coupled within participants, especially whether cervico-suboccipital rhythmicity relates to autonomic organization. The fourth is whether repeated stimulation-related signatures are associated with measurable changes in cerebrospinal fluid, lymphatic, glial, or immune markers.

The value of the hypothesis lies in this testability. If the pathway exists, bilateral or spatially organized stimulation should produce measurable changes in ocular motor activity, cervical or suboccipital muscle activity, cranio-cervical micro-motion, autonomic state, or indirect markers of autonomic-vascular and cerebrospinal fluid dynamics. If such signatures are absent, or if they do not differ from non-spatial or non-rhythmic controls, the hypothesis would require revision.

### 2.7 Mastoid and peri-auricular stimulation as a more direct upper-cervical route

Mastoid and peri-auricular stimulation provide a particularly relevant test case for the cervico-suboccipital sensorimotor convergence hypothesis. Unlike distal tactile stimulation, stimulation applied behind the ears or near the mastoid region is anatomically close to structures involved in vestibular-adjacent processing, cervical proprioception, upper cervical sensory input, local soft-tissue mechanoreception, and cranio-cervical muscle regulation.

This proximity allows mastoid or peri-auricular stimulation to engage the proposed cervico-suboccipital pathway more directly than distal tactile stimulation. In particular, mastoid-region stimulation provides a shorter route through local somatosensory input, vibration-sensitive mechanoreception, vestibular-adjacent activation, vestibulo-cervical pathways, and cervical proprioceptive mechanisms. The plausibility of this route is supported by vestibular and bone-conducted stimulation studies showing short-latency responses in neck musculature (Rosengren et al., 2010; Colebatch et al., 2016; Forbes et al., 2018), although specific suboccipital involvement remains an experimental prediction.

In this framework, mastoid or peri-auricular stimulation is not a departure from the broader bilateral stimulation framework, but a more anatomically targeted way to examine one of its possible physiological pathways. Visual bilateral stimulation may reach the cervico-suboccipital system through eye-head–neck coordination. Auditory stimulation may reach it through spatial orienting and head–neck preparation without direct mechanical contact with the head or neck. Distal tactile stimulation may reach it through longer routes involving somatosensory orienting, covert oculomotor preparation, postural preparation, and autonomic regulation. Mastoid or peri-auricular stimulation may engage the same downstream cervico-suboccipital target through a shorter upper-cervical route.

**Figure 1:**
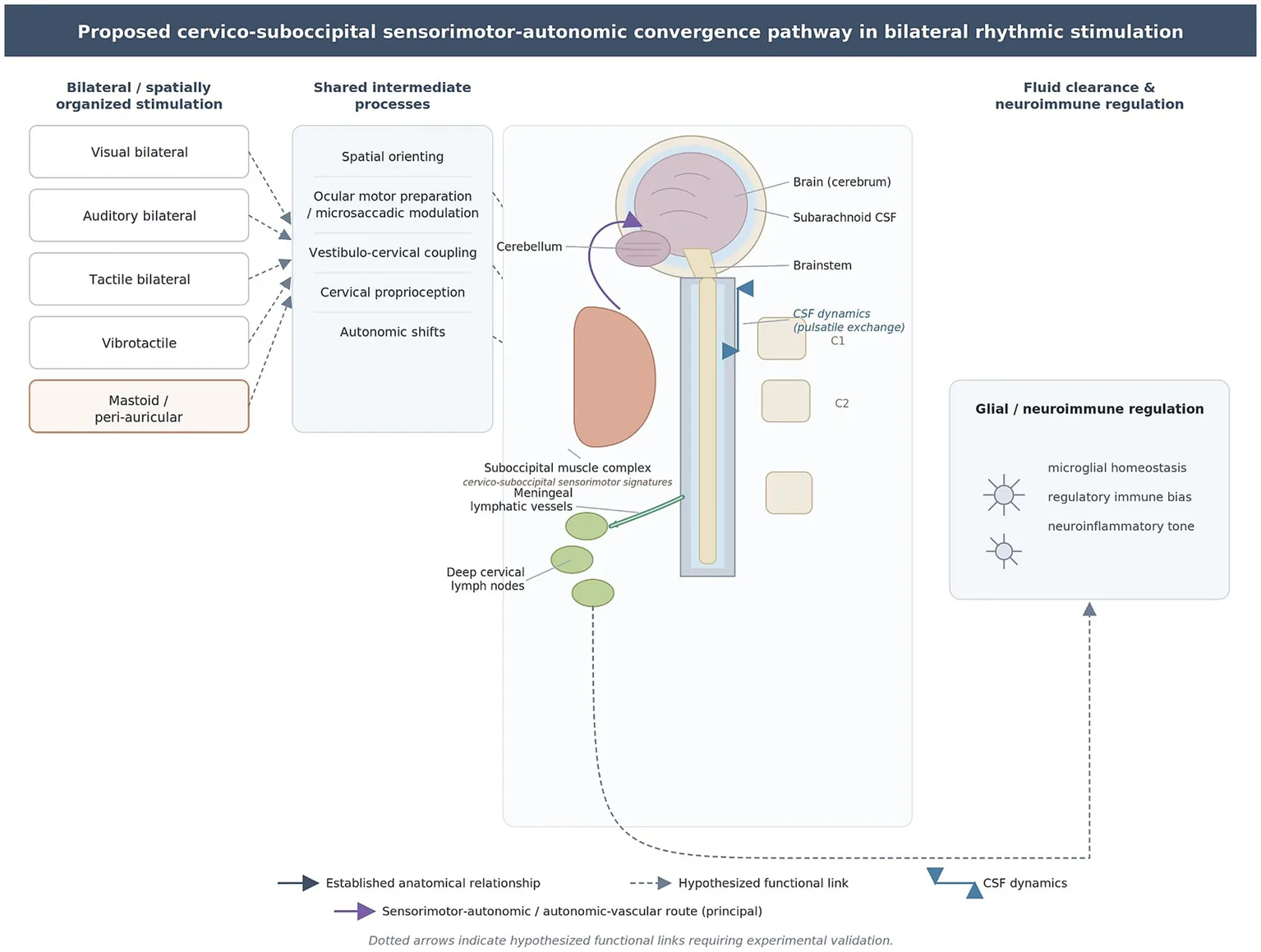
Proposed cervico-suboccipital sensorimotor-autonomic convergence pathway. The schematic illustrates the proposed upstream physiological component of the hypothesis rather than a complete model of all EMDR effects. Different forms of bilateral or spatially organized rhythmic stimulation are shown as engaging shared early processes, including orienting, ocular motor preparation, vestibulo-cervical coupling, cervical proprioception, and autonomic shifts. These processes converge on the cervico-suboccipital system, where the suboccipital muscle complex functions as a dense proprioceptive interface for gaze, head-position, vestibular, and cervical regulation. The principal route shown is a sensorimotor-autonomic and autonomic-vascular route: proprioceptive and autonomic changes influence brainstem autonomic regulation, vagal efferent output, vascular tone, arterial pulsatility, CSF secretion, and CSF/glymphatic exchange. Downstream, CSF exchange with meningeal lymphatic vessels and drainage toward deep cervical lymph nodes is shown as a slower hypothetical route through which repeated stimulation-related autonomic-vascular modulation may interact with glial state, immune surveillance, regulatory immune mechanisms, and neuroinflammatory tone. The possible relevance of spatial alternation is shown as a testable stimulation parameter rather than as proof of strict left–right specificity. Dotted arrows indicate hypothesized functional links requiring empirical testing and do not represent a proven causal chain.

The immediate value of this distinction is experimental. If mastoid or peri-auricular stimulation produces stronger or more reliable cervico-suboccipital signatures than distal tactile stimulation after controlling for passive mechanical artifact, it would help clarify whether anatomical proximity to the upper cervical region is functionally meaningful. If auditory bilateral stimulation also produces a stimulation-locked cervico-suboccipital signature despite the absence of direct mechanical contact, it would support a neurally mediated convergence pathway. If no such differences or signatures are observed, the hypothesis would require refinement (see Figure 1).

### 2.8 Integration with autonomic, meningeal lymphatic, and neuroimmune pathways

The downstream part of the proposed model depends on the connection between cerebrospinal fluid, meningeal lymphatic vessels, and cervical lymph nodes. Meningeal lymphatic vessels drain cerebrospinal fluid, macromolecules, immune cells, and brain-derived antigens toward cervical lymph nodes, including deep cervical lymph nodes, thereby linking fluid clearance with immune surveillance and neuroimmune regulation (Louveau et al., 2015; Raper et al., 2016; Rustenhoven and Kipnis, 2022; Salvador et al., 2024; Kipnis et al., 2025).

The proposed downstream interface is autonomic-vascular rather than directly mechanical. Rhythmic ocular, tactile, auditory, vestibulo-cervical, or peri-auricular input may modulate cervico-suboccipital proprioceptive afference and brainstem autonomic integration. A parasympathetic shift measured by heart rate variability would reflect vagal efferent output, but the relevant sensory input need not be vagal. In this sense, the vagus is treated here primarily as an output arm of autonomic regulation rather than as the peripheral afferent route from the stimulated region. Autonomic and vascular changes can then influence cerebral vascular tone, arterial pulsatility, and CSF secretion or exchange. Arterial pulsation has been implicated as an important driver of paravascular CSF-interstitial fluid exchange in the murine brain (Iliff et al., 2013), and *in vivo* studies have shown that CSF flow in perivascular spaces is pulsatile and closely coupled to arterial wall motion and blood-pressure-related vascular dynamics (Mestre et al., 2018). In addition, sympathetic and neuromodulatory influences on choroid plexus function support a link between autonomic or neuromodulatory state and CSF production (Lindvall et al., 1978; Edelbo et al., 2025).

This framing links the present article to the previously proposed neuroimmune hypothesis. The earlier model focused on the downstream possibility that repeated bilateral stimulation could interact with meningeal lymphatic drainage, deep cervical lymph node immune surveillance, and regulatory T-cell-centered neuroimmune modulation (Milovanov, 2026). The present article addresses the upstream question: how might bilateral or spatially organized rhythmic stimulation generate the autonomic and sensorimotor state that could feed into that slower pathway? Thus, the regulatory T-cell-centered mechanism is not re-established here; it is treated as a hypothetical downstream interface developed in the earlier article.

This interface should not be described as a simple mechanical pump. Computational work has cautioned that arterial pulsations drive oscillatory CSF motion without necessarily producing directional pumping, and that net directional movement depends on pressure gradients or other system-level conditions (Kedarasetti et al., 2020). Accordingly, the present hypothesis is framed in terms of autonomic-vascular modulation, CSF dynamics, CSF exchange, and downstream meningeal lymphatic drainage, rather than direct pumping of fluid by external stimulation.

Animal studies support the functional importance of this pathway. Impairment of meningeal lymphatic drainage can aggravate pathology in models of neurodegeneration. In Alzheimer’s disease models, meningeal lymphatic dysfunction has been associated with worsened amyloid pathology and age-associated cognitive decline (Da Mesquita et al., 2018). In a Parkinson’s disease model, blocking meningeal lymphatic drainage aggravated Parkinson’s-like pathology in mice overexpressing mutated alpha-synuclein (Zou et al., 2019). These findings support the biological relevance of the drainage pathway, although they do not by themselves show that external rhythmic stimulation can improve drainage or clinical outcome.

More directly relevant to the present hypothesis are studies in which meningeal lymphatic function was enhanced or modulated. VEGF-C-related enhancement of lymphatic drainage has been reported to promote drainage of brain-derived fluids, modulate neuroinflammation, and improve neurological outcome in a mouse model of ischemic stroke (Boisserand et al., 2024). Non-invasive near-infrared modulation of meningeal lymphatics has also been reported to improve lymphatic drainage, reduce Alzheimer’s disease-associated pathology, decrease neuroinflammation and neuronal damage, and improve cognition in aged and Alzheimer’s disease mouse models (Wang et al., 2024). These studies suggest that meningeal lymphatic function can influence neuroinflammatory state and behaviorally relevant outcomes in animal models.

The relevance of these studies to the present model is specific. They do not demonstrate that mastoid or peri-auricular stimulation induces cervico-suboccipital signatures in humans, nor do they establish that such signatures alter cerebrospinal fluid flow or enhance meningeal lymphatic drainage. They do, however, support the biological plausibility of a slower autonomic-vascular, cerebrospinal fluid, meningeal lymphatic, and cervical lymph node pathway through which repeated stimulation-related physiological changes could, in principle, interact with glial and neuroimmune regulation.

This pathway is also potentially relevant to neurodevelopmental conditions, although evidence remains preliminary. In BTBR autism-like mice, altered meningeal immunity and changes in brain-draining lymph nodes have been reported, including reduced regulatory T cell population and function in the meninges and brain-draining lymph nodes (Uddin et al., 2022). These findings do not provide clinical evidence for stimulation-based treatment in autism. They support the more limited idea that meningeal immune compartments and brain-draining lymph nodes participate in neuroimmune processes relevant to behavior in animal models.

This point is relevant to the origin of the present hypothesis. Repeated bilateral stimulation of tissues and fascia near the mastoid region was clinically observed by the author in children with autism spectrum disorder, with changes in regulation, engagement, communication, or behavioral organization also noted by parents, educators, or social workers. These observations were not collected under standardized research conditions and cannot establish efficacy or mechanism. They are included here as hypothesis-generating observations that motivated the question of whether repeated mastoid-region stimulation might produce measurable cervico-suboccipital, autonomic, vascular, fluid-dynamic, lymphatic, or neuroimmune signatures.

Thus, the extended model adds an upstream sensorimotor-autonomic layer to the previously proposed neuroimmune model. It proposes the following chain: repeated spatially organized rhythmic stimulation generates ocular, vestibulo-cervical, proprioceptive, autonomic, and cervico-suboccipital signatures; these signatures interact with brainstem autonomic regulation and vagal efferent output; autonomic-vascular modulation influences vascular tone, arterial pulsatility, CSF secretion or exchange, and physiological conditions relevant to meningeal lymphatic drainage; and repeated downstream interactions with cervical lymph node immune surveillance may contribute, hypothetically, to slower glial and regulatory immune modulation. This remains a testable framework rather than an established causal chain.

## 3 Experimental and methodological implications

The methodological implications are organized as staged tests. The first and most feasible level concerns a proximal cervico-suboccipital-autonomic coupling signature that can be measured with non-invasive methods. More distal CSF, vascular, meningeal lymphatic, and neuroimmune outcomes require later and more demanding experiments.

### 3.1 A first testable experiment: cervico-suboccipital-autonomic coupling

The first empirically testable level of the model is the proximal cervico-suboccipital-autonomic coupling hypothesis. This hypothesis does not require immediate measurement of CSF or meningeal lymphatic flow. It predicts that spatially organized rhythmic stimulation, including non-ocular bilateral stimulation, produces a measurable cervico-suboccipital physiological signature and that this signature is coupled to autonomic organization.

The novel outcome explained by the model is not a generic autonomic calming response. Such a response could be explained by working-memory load, nonspecific orienting, relaxation, or general arousal models. The distinctive prediction of the present convergence hypothesis is a cross-system coupling pattern: spatial rhythmic stimulation should organize cervical or suboccipital activity and autonomic regulation together. Two primary contrasts are especially important. First, auditory bilateral stimulation provides a critical discriminating condition. Because it involves no direct mechanical contact with the head or neck, any stimulation-locked cervico-suboccipital signature would be difficult to attribute to passive mechanical transmission and would instead support a neurally mediated convergence pathway involving orienting, ocular motor preparation, vestibulo-cervical coupling, or cervico-suboccipital proprioceptive engagement. Second, mastoid or peri-auricular stimulation should produce a stronger cervico-suboccipital rhythmic signature than distal tactile stimulation under matched conditions, but this contrast must be interpreted with explicit control for possible mechanical vibration or conduction artifacts. Within participants, conditions producing stronger cervico-suboccipital rhythmic signatures should also produce stronger autonomic organization. Thus, the key prediction is not simply that stimulation changes autonomic state, but that autonomic change is coupled to the strength of the cervico-suboccipital signature.

A feasible first-stage experiment would use a within-subject randomized crossover design. Each participant would receive several stimulation conditions matched for frequency, duration, and perceived intensity, with perceived intensity calibrated individually before the experiment using a brief psychophysical adjustment procedure. The stimulation conditions could include visual bilateral stimulation, auditory bilateral stimulation delivered through headphones, mastoid or peri-auricular bilateral stimulation, distal tactile stimulation, simultaneous bilateral stimulation, random stimulation, and sham or rest control. The primary quantitative measures would be cervical or suboccipital surface electromyography, spectral power at the stimulation frequency, coherence with the stimulation rhythm, and head–neck micro-motion measured by inertial sensors or optical tracking. For mastoid or peri-auricular stimulation, accelerometry at or near the recording site, together with latency, phase, and coherence analyses, should be used to distinguish neurally mediated rhythmicity from passive mechanical artifact. Secondary measures would include eye tracking measures such as microsaccade rate, gaze stability, directionality, and phase-locking to the stimulation rhythm, together with autonomic measures such as heart rate variability, respiratory coherence, electrodermal activity, pupillary dynamics, and peripheral pulse measures.

The analysis should explicitly control for cardiac and respiratory oscillations, because these are strong physiological rhythms that could overlap with stimulation-related signals. This can be addressed by recording respiration and pulse simultaneously, selecting stimulation frequencies that are distinguishable from dominant cardiac and respiratory frequencies when possible, and using regression, coherence, or cross-spectral analyses to separate stimulation-locked signatures from cardiorespiratory activity.

This first-stage study is feasible with non-invasive and relatively accessible methods: a controlled rhythmic stimulation protocol, surface electromyography, inertial or optical head–neck motion tracking, heart-rate variability monitoring, electrodermal recording, respiratory monitoring, and, where available, eye tracking or pupillometry. A proof-of-mechanism study could initially be conducted in approximately 20–30 healthy adults using a within-subject crossover design, before moving to clinical populations or more complex imaging-based studies.

The hypothesis is falsifiable. Most critically, it would be weakened if auditory bilateral stimulation produced no stimulation-locked cervico-suboccipital or head–neck rhythmic signature, because this condition provides the cleanest test of neurally mediated convergence without direct mechanical contact with the head or neck. It would also be weakened if mastoid or peri-auricular stimulation did not produce a stronger cervico-suboccipital rhythmic signature than distal tactile stimulation under matched conditions after controlling for mechanical artifact; if non-ocular spatial stimulation produced autonomic effects without any measurable cervico-suboccipital or head–neck rhythmic signature; if the strength of cervico-suboccipital rhythmicity did not relate to autonomic organization within participants; or if random, simultaneous, and spatially organized stimulation produced equivalent physiological signatures. CSF, vascular, and meningeal lymphatic outcomes represent a later and more demanding level of testing, using methods such as transcranial Doppler, vascular ultrasound, phase-contrast MRI of CSF flow, venous outflow assessment, or animal models. Thus, the first test of the hypothesis concerns a measurable cross-system physiological coupling pattern, while the CSF-lymphatic component remains a distal experimental target.

### 3.2 Eye tracking and microsaccade analysis

If non-visual bilateral stimulation engages the oculomotor system through covert spatial orienting, then alternating tactile, auditory, vibrotactile, or mastoid-region stimulation should produce measurable changes in microsaccades, gaze bias, fixation stability, or ocular motor preparation. High-resolution eye tracking could be used while participants maintain central fixation and receive alternating non-visual stimulation. This would allow investigators to examine whether microsaccade direction, timing, rate, or amplitude is modulated by the side and timing of stimulation.

The key prediction is not the presence of large visible eye movements. The model predicts subtle rhythmic or lateralized ocular signatures time-locked to the stimulation pattern. These should be compared with unilateral, non-spatial, randomized, and non-rhythmic control conditions. If non-visual bilateral stimulation produces lateralized microsaccadic modulation, this would support the idea that spatial attention and ocular preparation participate in the physiological response to bilateral stimulation (Hafed and Clark, 2002; Engbert and Kliegl, 2003; Laubrock et al., 2005).

At the same time, the interpretation should remain cautious. Microsaccades are related to covert attention, but this relationship is not obligatory and may depend on task demands, fixation instructions, sensory salience, and timing (Casteau and Smith, 2019; Liu et al., 2024). Therefore, a negative result in one paradigm would not necessarily rule out the broader model, but it would help define the conditions under which ocular orienting is or is not involved.

### 3.3 Cervical and suboccipital electromyography

If rhythmic spatial stimulation engages the cervico-suboccipital system, it should produce measurable changes in cervical or suboccipital muscle activity even when overt head movement is absent. Electromyographic studies could compare visual bilateral stimulation, auditory bilateral stimulation, distal tactile stimulation, vibrotactile stimulation, and mastoid or peri-auricular stimulation while recording from accessible cervical and upper cervical muscle regions.

Because the deepest suboccipital muscles are difficult to record non-invasively, initial studies may use surface electromyography over cervical paraspinal or upper cervical regions as indirect proxies. More precise studies could use high-density electromyography, ultrasound-guided approaches, or paradigms designed to minimize activity from larger superficial muscles. The main prediction is not strong activation, but rhythmic modulation time-locked to the stimulation pattern.

Vestibular-evoked myogenic potential and related vestibular stimulation paradigms provide a useful methodological precedent because they show that vestibular and bone-conducted stimuli can evoke measurable responses in neck musculature (Rosengren et al., 2010; Colebatch et al., 2016; Forbes et al., 2018). For the present hypothesis, mastoid or peri-auricular stimulation would be especially informative if it produced stronger or more reliable cervical signatures than distal tactile stimulation matched for frequency and perceived intensity.

### 3.4 Head–neck micro-motion and cranio-cervical sensorimotor state

The hypothesis also predicts subtle rhythmic head–neck micro-motion or changes in cranio-cervical sensorimotor state. These changes may be too small to observe visually. They could be measured using inertial measurement units, accelerometry, high-resolution motion capture, force-sensitive head supports, ultrasound-based tissue motion measures, or other methods sensitive to low-amplitude movement and rhythmic oscillation.

A useful design would compare bilateral spatial stimulation with control stimulation matched for sensory intensity but lacking left–right organization or rhythmic predictability. If the proposed pathway is active, bilateral rhythmic stimulation should produce more structured cranio-cervical micro-motion signatures than unilateral, randomized, or non-spatial stimulation.

Methodologically, stimulation-related micro-motion will need to be separated from respiration, cardiac pulsatility, swallowing, postural sway, and general body movement. Simple filtering may be insufficient because the expected stimulation-related rhythm could overlap with physiological rhythms. More appropriate approaches may include stimulation-locked averaging, coherence analysis, phase-locking analysis, regression using respiratory and electrocardiographic reference signals, and comparison with carefully matched control conditions.

### 3.5 Autonomic coupling

The cervico-suboccipital pathway may interact with autonomic regulation. Therefore, studies should combine ocular, cervical, and motion measures with autonomic markers such as heart rate variability, electrodermal activity, respiration, pupillary measures, peripheral temperature, and peripheral perfusion. These measures would help distinguish rapid bodily-autonomic effects from slower fluid-dynamic or neuroimmune hypotheses.

As outlined in Section 3.1, autonomic measures should be interpreted in relation to stimulation-locked cervico-suboccipital and ocular signatures rather than as isolated calming effects. The central prediction is coupling: conditions producing stronger stimulation-locked electromyographic, micro-motion, or ocular signatures should also show stronger autonomic organization.

Autonomic measures may also help identify individual differences. Baseline arousal, anxiety state, respiratory pattern, vestibular sensitivity, sensory defensiveness, and cervical muscle tone may influence whether rhythmic stimulation produces calming, activating, or mixed effects. This may be especially important in neurodevelopmental populations, where sensory processing and autonomic regulation can differ substantially from typical adult samples.

### 3.6 Cerebrospinal fluid and meningeal lymphatic proxies

Direct measurement of cerebrospinal fluid dynamics and meningeal lymphatic function in humans remains challenging. Nevertheless, the model generates experimentally approachable predictions. In humans, possible methods include magnetic resonance imaging-based measures of cerebrospinal fluid flow, cranio-cervical fluid pulsatility, intracranial compliance proxies, glymphatic or perivascular flow markers, and indirect measures of cervical lymphatic drainage (Ringstad and Eide, 2018; Salvador et al., 2024; Kipnis et al., 2025). In animal models, tracer-based studies, pressure or flow measurements, lymphatic imaging, and tissue-level neuroimmune assays may allow more direct testing.

The strongest evidence would come from linked multimodal findings. For example, a study could test whether repeated spatial rhythmic stimulation produces ocular and cervico-suboccipital signatures; whether these signatures are associated with changes in cerebrospinal fluid pulsatility or cranio-cervical fluid dynamics; and whether repeated stimulation is associated with changes in meningeal lymphatic drainage, cervical lymph node immune markers, glial activation, or inflammatory tone.

At present, this sequence remains a research program rather than an established mechanism. However, it is experimentally meaningful because each link can be tested separately. A finding that stimulation alters autonomic or subjective state without any measurable fluid-dynamic signature would support a proximal autonomic interpretation but weaken the proposed slower cerebrospinal fluid pathway. Conversely, a finding that repeated stimulation alters cranio-cervical fluid-dynamic or lymphatic markers in association with autonomic and cervico-suboccipital signatures would provide stronger support for the proposed sensorimotor-autonomic-neuroimmune route.

### 3.7 Modality comparison

A key prediction of the model is that different forms of bilateral stimulation show partial convergence but not identical physiological signatures. Visual bilateral stimulation should engage ocular motor and eye-head–neck systems most directly. Auditory stimulation is especially important as a non-contact test of neurally mediated convergence through spatial orienting, ocular motor preparation, vestibulo-cervical coupling, and autonomic regulation. Distal tactile stimulation engages the pathway more indirectly through somatosensory orienting and postural preparation. Mastoid or peri-auricular stimulation produces a more anatomically proximal cervico-suboccipital signature through local somatosensory or vibrotactile input, vestibular-adjacent pathways, and cervical proprioceptive mechanisms, but this condition requires explicit control for passive mechanical artifact.

A within-participant modality comparison would therefore be particularly informative. Each participant could receive visual, auditory, distal tactile, vibrotactile, and mastoid or peri-auricular stimulation under matched conditions. Measures would include high-resolution eye tracking, microsaccade analysis, cervical electromyography, head–neck micro-motion, heart rate variability, respiration, electrodermal activity, and subjective state.

The model would be supported if different modalities produce partially overlapping ocular, cervical, and autonomic signatures, with auditory stimulation showing a stimulation-locked cervico-suboccipital signature despite the absence of direct mechanical contact, and with mastoid or peri-auricular stimulation producing a stronger or more direct cervico-suboccipital pattern than distal tactile stimulation after controlling for mechanical artifact. The model would need refinement if all modalities produce only general arousal changes without spatially organized ocular or cervical signatures, if auditory stimulation produces no cervico-suboccipital signature, or if mastoid-region stimulation does not differ from distal tactile stimulation when sensory intensity, attentional demand, and mechanical artifact are controlled.

### 3.8 Repeated-session studies and clinical translation

Because the slower part of the model concerns repeated physiological modulation, single-session studies are necessary but not sufficient. Acute experiments can identify ocular, cervical, autonomic, and micro-motion signatures, but repeated-session studies are needed to test whether these signatures are stable, cumulative, and associated with changes in regulation, behavior, or neuroimmune markers.

In clinical or translational studies, stimulation protocols should be standardized in frequency, duration, intensity, anatomical placement, posture, and instructions. Outcome measures should include both behavioral and physiological indices. In autism spectrum disorder, for example, pilot studies could assess tolerability, comfort, sensory acceptance, regulation, engagement, communication, parent and educator reports, and clinician-rated change, while also recording autonomic and sensorimotor measures. Such studies should not rely only on subjective improvement, because the central question is whether repeated stimulation produces measurable physiological signatures.

A staged approach is appropriate. First, healthy adult participants can be used to test whether the physiological signatures exist under controlled conditions. Second, repeated-session studies can examine whether signatures remain stable and whether they change with habituation, relaxation, or learning. Third, carefully designed pilot studies in relevant clinical populations can test feasibility, tolerability, and preliminary associations between physiological signatures and behavioral outcomes.

### 3.9 Boundary conditions and falsifiability

The hypothesis should be formulated in a way that allows revision or rejection. The proximal convergence hypothesis would be weakened most directly if auditory bilateral stimulation produces no stimulation-locked cervico-suboccipital or head–neck rhythmic signature, because this condition provides the cleanest test of neurally mediated convergence without direct mechanical contact. It would also be weakened if observed activity does not differ from unilateral, randomized, simultaneous, or non-spatial control stimulation; if autonomic effects occur independently of ocular or cervical signatures; or if mastoid/peri-auricular stimulation does not produce stronger or more direct cervico-suboccipital signatures than matched distal stimulation after controlling for mechanical artifact.

It would also be weakened if repeated stimulation fails to produce any association between cervico-suboccipital signatures and fluid-dynamic, lymphatic, glial, immune, or behavioral measures. Such findings would not invalidate EMDR, working-memory accounts, orienting-response models, autonomic explanations, or stochastic resonance models. They would specifically challenge the proposed sensorimotor-autonomic convergence pathway.

The converse pattern would strengthen the model: time-locked ocular and cervical signatures during spatial rhythmic stimulation; modality-specific differences consistent with anatomical route; coupling between cervico-suboccipital signatures and autonomic regulation; and, in repeated-session studies, associations with cerebrospinal fluid, meningeal lymphatic, cervical lymph node, glial, immune, or behavioral markers. This staged logic makes the hypothesis experimentally tractable even before the full pathway is demonstrated.

## 4 Clinical and translational implications

The main translational implication of the cervico-suboccipital sensorimotor convergence hypothesis is not that bilateral stimulation should be reduced to a bodily intervention. Rather, the hypothesis suggests that EMDR and related forms of rhythmic spatial stimulation should be studied as multi-level physiological procedures. Cognitive, emotional, autonomic, sensorimotor, and possibly fluid-dynamic or neuroimmune processes occur in parallel, and different stimulation modalities emphasize different levels of this system.

For EMDR research, this framework encourages a broader measurement strategy. Studies of bilateral stimulation often focus on symptom change, memory vividness, emotionality, or subjective distress. These are essential outcomes, but they may not capture the full physiological response to stimulation. If rhythmic spatial stimulation produces ocular, cervical, autonomic, or cranio-cervical signatures, these should be measured directly. Eye tracking, microsaccade analysis, cervical electromyography, head–neck micro-motion recording, respiration, heart rate variability, electrodermal activity, and pupillary measures could help distinguish cognitive load effects from bodily-autonomic and cervico-suboccipital effects.

The model also has implications for stimulation parameters. Frequency, rhythm regularity, spatial alternation, sensory modality, anatomical placement, intensity, posture, gaze condition, breathing state, and baseline arousal influence the physiological signature of stimulation. Future studies should therefore report stimulation parameters more precisely than is often done in clinical descriptions. This is especially important when comparing visual eye movements, auditory bilateral tones, distal tactile stimulation, vibrotactile stimulation, and mastoid or peri-auricular stimulation.

Mastoid and peri-auricular stimulation is particularly useful as an experimental probe. Because this region is close to the temporal bone, vestibular-adjacent structures, cervical proprioceptive systems, and suboccipital regulation, it can help test whether rhythmic stimulation engages the cervico-suboccipital pathway more directly than distal stimulation. This does not make mastoid stimulation a separate replacement for EMDR. It makes it a targeted route for examining one physiological component that can also be engaged more indirectly by other forms of bilateral stimulation.

The hypothesis also guides the development of physiologically monitored stimulation systems. Wearable or clinic-based devices could combine rhythmic stimulation with real-time recording of autonomic and sensorimotor signals. For example, stimulation could be paired with heart rate variability, respiration, electrodermal activity, peripheral temperature, accelerometry, or cervical muscle measures. Such systems would not need to assume in advance which stimulation pattern is optimal. Instead, they could be used experimentally to identify stimulation parameters associated with comfort, autonomic regulation, stable engagement, and measurable cervico-suboccipital rhythmicity.

Closed-loop systems are one possible future direction. If specific stimulation patterns are associated with improved autonomic organization or clearer cervico-suboccipital signatures, stimulation parameters could in principle be adjusted in response to physiological signals. This is useful for research on individual differences, because the same stimulation frequency or intensity does not produce the same response in all participants. Baseline arousal, sensory sensitivity, vestibular responsiveness, cervical muscle tone, age, posture, trauma state, and neurodevelopmental profile all influence the response.

Neurodevelopmental populations require special attention. In autistic individuals, sensory processing, motor control, vestibular responsiveness, autonomic regulation, and tolerance of bodily stimulation can differ substantially across individuals. Therefore, any translational work in autism spectrum disorder should begin with feasibility, tolerability, sensory acceptance, and safety. Early studies should prioritize structured protocols, parent and clinician reports, standardized behavioral measures, and physiological monitoring rather than broad claims of efficacy.

A staged translational pathway is appropriate. First, controlled studies in healthy adults can test whether different stimulation modalities produce the predicted ocular, cervical, autonomic, and micro-motion signatures. Second, repeated-session studies can examine whether these signatures are stable, cumulative, or associated with changes in autonomic regulation or subjective state. Third, pilot studies in clinical or neurodevelopmental populations can evaluate feasibility, tolerability, and preliminary associations between physiological signatures and behavioral outcomes. Only later would larger controlled clinical studies be justified.

The hypothesis also suggests that ordinary clinical variables have physiological importance. Head position, cervical tension, fixation state, breathing rhythm, posture, fatigue, anxiety level, and therapist instructions influence whether the proposed pathway is engaged. These variables are often treated as background conditions, but they partly determine the bodily response to rhythmic stimulation. Future protocols should therefore document or control them when comparing stimulation modalities.

Overall, the translational value of the model lies in making bilateral stimulation more measurable. Rather than treating visual, auditory, tactile, vibrotactile, and mastoid stimulation as interchangeable forms of “bilateral input,” the model encourages direct comparison of their physiological signatures. This may help clarify which effects are related to cognitive load, which are related to orienting and autonomic regulation, and which, if any, are related to slower cervico-suboccipital, cerebrospinal fluid, meningeal lymphatic, or neuroimmune pathways.

## 5 Limitations

The cervico-suboccipital sensorimotor convergence hypothesis remains theoretical. Its strength lies in anatomical plausibility, physiological coherence, and experimental testability, not in direct empirical confirmation. At present, there is no direct evidence that EMDR or other forms of bilateral rhythmic stimulation produce functionally meaningful cervico-suboccipital-autonomic signatures, alter cerebrospinal fluid dynamics, enhance meningeal lymphatic drainage, or modulate neuroimmune processes in humans through the pathway proposed here.

The model contains several separable links, each requiring independent testing. These include the possible recruitment of covert ocular orienting during non-visual bilateral stimulation; the coupling between ocular preparation and cervical or suboccipital muscle activity; the coupling between cervico-suboccipital rhythmicity and autonomic organization; the influence of autonomic-vascular state on cerebrospinal fluid dynamics; and the possible downstream interaction with meningeal lymphatic exchange, cervical lymph node immune surveillance, glial regulation, regulatory T-cell-related mechanisms, and neuroimmune tone. Evidence for one link would not automatically establish the entire pathway.

The proposed role of covert ocular orienting also requires careful qualification. Microsaccades and ocular motor preparation can reflect covert spatial attention, but this relationship is context-dependent rather than obligatory (Hafed and Clark, 2002; Engbert and Kliegl, 2003; Casteau and Smith, 2019; Liu et al., 2024). Therefore, non-visual bilateral stimulation should not be assumed to produce microsaccadic modulation in every paradigm or individual. Eye tracking studies are needed to determine whether tactile, auditory, vibrotactile, or mastoid/peri-auricular stimulation reliably produces ocular motor signatures, and whether these signatures are functionally related to cervical or suboccipital activity.

The role of cerebrospinal fluid, meningeal lymphatics, and cervical lymph nodes remains a distal hypothesis. The present manuscript does not establish that bilateral stimulation alters CSF dynamics or lymphatic drainage. It proposes a narrower upstream pathway in which rhythmic spatial stimulation may generate measurable cervico-suboccipital and autonomic signatures that could, over repeated sessions, interface with autonomic-vascular and meningeal lymphatic mechanisms proposed in the earlier neuroimmune model.

Measurement presents an additional challenge. The deepest suboccipital muscles are small, deep, and difficult to record non-invasively. Surface electromyography can capture mixed signals from superficial cervical muscles and does not isolate the specific deep muscles most relevant to upper cervical proprioception. More precise approaches, including high-density electromyography, ultrasound-guided assessment, motion tracking, or translational animal studies, are likely required. Similarly, head–neck micro-motion must be separated from respiration, cardiac pulsatility, swallowing, postural sway, and general movement.

A further limitation concerns the distinction between proprioceptive activation and downstream physiological effect. Evidence for eye-neck or cervico-ocular coupling supports the possibility that rhythmic ocular or spatial stimulation may modulate cervical and suboccipital activity, but such activity does not by itself demonstrate changes in CSF dynamics, lymphatic drainage, or immune state. Future studies should therefore measure cervical electromyography, suboccipital or head–neck micro-motion, autonomic state, vascular pulsatility, and CSF or lymphatic proxies simultaneously, rather than inferring one level from another.

Cerebrospinal fluid dynamics and meningeal lymphatic function are also difficult to measure directly in humans. They are influenced by respiration, cardiac pulsatility, sleep–wake state, posture, vascular dynamics, intracranial compliance, and autonomic state. Even if changes in cerebrospinal fluid pulsatility or lymphatic drainage are observed during or after rhythmic stimulation, careful control conditions will be needed to determine whether these changes are specifically related to spatially organized stimulation, cervical micro-motion, autonomic regulation, or more general physiological state changes.

The downstream neuroimmune part of the model is supported mainly by animal literature showing that meningeal lymphatic function can influence neuroinflammation and behaviorally relevant outcomes (Da Mesquita et al., 2018; Zou et al., 2019; Boisserand et al., 2024; Wang et al., 2024). These studies support biological plausibility, but they do not demonstrate that external rhythmic stimulation can modulate meningeal lymphatic drainage in humans. They also do not establish that mastoid or peri-auricular stimulation can improve cognitive, emotional, or behavioral outcomes through a lymphatic mechanism.

It is also important to distinguish the proposed slower pathway from rapid effects during a single EMDR session. Acute reductions in distress, vividness, emotionality, or arousal are better explained by working-memory taxation, dual attention, orienting responses, autonomic regulation, expectancy, therapeutic context, or other established mechanisms. The present model is not intended to replace those accounts. It proposes an additional repeated-stimulation pathway that is relevant over a slower physiological timescale.

EMDR itself is a complex therapeutic procedure and cannot be reduced to bilateral stimulation alone. Memory activation, therapist-client interaction, dual attention, expectancy, affect regulation, cognitive reappraisal, and therapeutic safety all contribute to clinical outcome. Therefore, even if cervico-suboccipital signatures are demonstrated during bilateral stimulation, their clinical relevance would still need to be tested separately.

Finally, the model does not apply equally across individuals, stimulation modalities, or clinical populations. Cervical anatomy, vestibular sensitivity, ocular motor patterns, posture, muscle tone, age, trauma state, autonomic baseline, sensory processing, and neurodevelopmental profile influence whether the proposed pathway is engaged. In autistic individuals and other neurodevelopmental populations, sensory responsiveness, motor control, vestibular processing, and autonomic regulation can vary widely. These differences should be treated not as noise, but as important variables for future research.

These limitations define the empirical boundaries of the hypothesis. They do not remove the rationale for the model, but they require that it be tested stepwise: first by demonstrating ocular, cervical, autonomic, and head–neck signatures; then by determining whether these signatures relate to cerebrospinal fluid or lymphatic measures; and only later by asking whether repeated stimulation has clinically meaningful effects in specific populations.

## 6 Conclusion and future directions

This article proposes that cervico-suboccipital sensorimotor convergence represents a testable upstream autonomic pathway for bilateral or spatially organized rhythmic stimulation. The model does not reduce EMDR to a bodily process and does not treat cerebrospinal fluid or meningeal lymphatic mechanisms as explanations for rapid within-session change. Instead, it distinguishes three partially overlapping levels: rapid cognitive-attentional effects, rapid bodily-autonomic effects, and slower repeated-stimulation effects in which cervico-suboccipital and autonomic-vascular signatures may interface with cerebrospinal fluid dynamics, meningeal lymphatic exchange, cervical lymph node immune surveillance, glial regulation, and regulatory immune mechanisms.

The present hypothesis broadens the question beyond EMDR alone. EMDR remains an important clinical context because rhythmic bilateral stimulation is a central procedural feature. However, the broader physiological question concerns whether rhythmic spatial stimulation, whether visual, auditory, tactile, vibrotactile, or mastoid/peri-auricular, can generate measurable ocular, vestibulo-cervical, proprioceptive, autonomic, and cervico-suboccipital signatures. These signatures differ by modality and anatomical route while still partly converging on shared orienting, gaze-postural, and cranio-cervical systems.

A key implication of the model is that non-visual bilateral stimulation should not be studied only as a cognitive distractor or dual-attention task. Alternating tactile, auditory, vibrotactile, or mastoid-region stimulation also engages covert spatial orienting, microsaccadic modulation, ocular motor preparation, cervical proprioception, vestibulo-cervical coupling, and autonomic regulation. These pathways provide measurable entry points for testing whether different forms of bilateral stimulation share a common eye-neck-suboccipital component.

Meningeal lymphatic and cervical lymph node studies suggest a biologically plausible downstream route linking cerebrospinal fluid exchange, immune surveillance, neuroinflammation, and behaviorally relevant outcomes in animal models. The present manuscript does not claim that this downstream pathway has been demonstrated for bilateral stimulation in humans. Rather, it proposes an upstream sensorimotor-autonomic route that could feed into the previously proposed neuroimmune model and can be tested independently before more demanding CSF, lymphatic, or immune measures are attempted.

Future research should therefore proceed stepwise. The first priority is to determine whether different forms of rhythmic spatial stimulation produce time-locked ocular, cervical, suboccipital, autonomic, or head–neck signatures under controlled conditions. The second priority is to compare stimulation modalities directly, including visual eye movements, auditory stimulation, distal tactile stimulation, vibrotactile stimulation, and mastoid or peri-auricular stimulation. The third priority is to determine whether repeated stimulation-related signatures are associated with cerebrospinal fluid, meningeal lymphatic, cervical lymph node, glial, immune, or behavioral measures.

If supported, the cervico-suboccipital sensorimotor convergence hypothesis would help explain why different forms of bilateral or spatially organized rhythmic stimulation produce overlapping regulatory effects despite entering through different sensory channels. It would also guide the development of more physiologically informed stimulation protocols, including protocols that monitor ocular, cervical, autonomic, and motion signals during stimulation. If not supported, the hypothesis would still be useful by narrowing the search for mechanisms and clarifying which physiological pathways are not necessary for the effects of bilateral stimulation.

In either case, the model encourages a more integrated research approach. EMDR and related rhythmic stimulation procedures should be studied not only through cognitive and clinical outcomes, but also through ocular motor physiology, cervico-suboccipital sensorimotor physiology, autonomic regulation, cerebrospinal fluid dynamics, meningeal lymphatic function, and neuroimmune models. This integrated approach helps distinguish rapid cognitive and autonomic effects from slower repeated-stimulation mechanisms, and opens a more precise experimental path for studying how rhythmic spatial stimulation interacts with brain–body regulation.

## Data Availability

The original contributions presented in the study are included in the article/supplementary material, further inquiries can be directed to the corresponding author.
